# ACR-SA: attention-based deep model through two-channel CNN and Bi-RNN for sentiment analysis

**DOI:** 10.7717/peerj-cs.877

**Published:** 2022-03-17

**Authors:** Marjan Kamyab, Guohua Liu, Abdur Rasool, Michael Adjeisah

**Affiliations:** 1 School of Computer Science and Technology, Donghua University, Shanghai, China; 2 Shenzhen College of Advanced Technology, University of Chinese Academy of Sciences, Beijing, China; 3 Shenzhen Key Laboratory for High Performance Data Mining, Shenzhen Institutes of Advanced Technology, Chinese Academy of Sciences, Shenzhen, China; 4 College of Mathematics and Computer Science, Zhejiang Normal University, Zhejiang, Jinhua, China

**Keywords:** Deep learning, Convolutional neural network, Bi-direction recurrent neural network, Attention mechanism, Social media

## Abstract

Convolutional Neural Networks (CNN) and Recurrent Neural Networks (RNN) have been successfully applied to Natural Language Processing (NLP), especially in sentiment analysis. NLP can execute numerous functions to achieve significant results through RNN and CNN. Likewise, previous research shows that RNN achieved meaningful results than CNN due to extracting long-term dependencies. Meanwhile, CNN has its advantage; it can extract high-level features using its local fixed-size context at the input level. However, integrating these advantages into one network is challenging because of overfitting in training. Another problem with such models is the consideration of all the features equally. To this end, we propose an attention-based sentiment analysis using CNN and two independent bidirectional RNN networks to address the problems mentioned above and improve sentiment knowledge. Firstly, we apply a preprocessor to enhance the data quality by correcting spelling mistakes and removing noisy content. Secondly, our model utilizes CNN with max-pooling to extract contextual features and reduce feature dimensionality. Thirdly, two independent bidirectional RNN, *i.e*., Long Short-Term Memory and Gated Recurrent Unit are used to capture long-term dependencies. We also applied the attention mechanism to the RNN layer output to emphasize each word’s attention level. Furthermore, Gaussian Noise and Dropout as regularization are applied to avoid the overfitting problem. Finally, we verify the model’s robustness on four standard datasets. Compared with existing improvements on the most recent neural network models, the experiment results show that our model significantly outperformed the state-of-the-art models.

## Introduction

Nowadays, with extensive amounts of user-generated text on social media, sentiment analysis (SA) has become essential for NLP (Naseem et al., 2020). The sentiments involved in social networks are indeed sources for modeling business strategies to achieve the business goal. Although the amount of data in social media repositories increases exponentially, the traditional algorithms often fail to extract the sentiments from such big data.

Numerous studies have been conducted for sentiment analysis using a traditional classification based on manual feature engineering. Traditional methods do not present features that have massive weight. Usually, it focuses on the statically and word indicators, such as term frequency-inverse document frequency (TF-IDF). Researchers recently started to use deep learning (DL) approaches based on the distributed representation to deal with data specifications during training datasets, feature engineering, and other meeting problems on traditional techniques (Chen et al., 2020). DL approaches have been significantly implemented in multiple areas of NLP, such as family information extraction (Shi et al., 2019), behavior detection systems during the COVID-19 pandemic (Rosa et al., 2020), and emotion detection (Chen & Hao, 2020). These approaches revealed that it is improving accuracy and decreasing the prediction time. Neural networks (NN) such as CNN and RNN accomplished comparable results for the sentiment classification. Shi et al. (2019) proposed a model that improves aspect-based sentiment classification performance using CNN and can extract valuable features from the text data. However, CNN needs to rely on several convolution layers to extract higher features for capturing long-term dependencies because of the locality of convolutional and pooling (Zhang, Zhao & Lecun, 2015). RNN can overcome the mentioned issue due to a single layer that can handle long-term dependencies (Hassan & Mahmood, 2018). Long Short-Term Memory (LSTM) is an advanced RNN that uses three gates (input, forget, output). These gates help detect whether data in the previous state should be retrained or forgotten in the current state. Therefore, LSTM can address long-term information preservation and vanishing gradient text (Zhang et al., 2019). Similarly, Gate Recurrent Unit (GRU) is another variant of LSTM that has only two gates by consolidating the input gates and the forget gates. Nevertheless, the basic LSTM and GRU only scan in one sequence direction.

Bidirectional Long Short-Term Memory (BiLSTM) and Bidirectional Gated Recurrent Unit (BiGRU) are further developments that scan in both sequence directions and simultaneous access to forward and backward contexts (Li et al., 2020a). Abid et al. (2019) proposed joined architecture that used BiLSTM and BiGRU at first to capture long-term dependencies, and CNN utilized to reduce the loss and extract local information. The method achieved reliable classification accuracy on various benchmark datasets. However, their model did not consider the differences between the importance of different parts of sentences. Meantime, attention mechanisms have attracted the researcher’s concentration as it trains the model to learn better and let the network know where to perform its tasks. Studies showed that the attention-based mechanism positively impacts several NLP tasks because it trains the model to learn better and lets the network know where to perform its tasks. Zhou et al. (2017) proposed a BiLSTM attention-based model to extract features with a crucial classification effect. In comparison, Yang et al. (2016) proposed the hierarchical RNN method to learn attention weights based on the local context. Although these neural network models have been successful in sentiment analysis, this single attention mechanism is often used to capture important input information. However, under different representation subspaces, the same words may express different levels of emotion in different places so that all information constitutes the overall meaning of the entire input sequence. Nevertheless, there is a problem with the input sequence, and the file is eventually decoded to a specific length vector no matter how long the input text sequence is. Our previous work attention-based model utilizes CNN with LSTM (ACL) for sentiment classification (Kamyab, Liu & Adjeisah, 2021). In this model CNN utilizes to extract contextual features and bidirectional LSTM to capture long-term dependencies. Attention mechanism employed at the output of CNN to give different focus on the features. However, this model only works for short comments.

We propose an attention-based deep model using two-channel CNN and Bi-RNN sentiment analysis (ACR-SA) to solve the existing issues. The model considers forward and backward contextual dependencies, extracts the most critical feature, and pays attention to each token’s importance. It is designed to detect both long and short text polarity and work for small and large datasets. In the proposed model, we employed GloVe (https://nlp.stanford.edu/projects/glove/) word embedding as input for the weights and lengths to feed the two parallel CNN networks. We used a zero-padding strategy to make the model suitable for long and short text input text and Gaussian noise and Dropout were used as regularization in the input layer to combat overfitting. CNN is used to learn high-level feature context from input representation. Two independent BiLSTM and BiGRU extract the contextual information from the feature-generated CNN layers and consider forward and backward contextual dependencies to perform the sentiment analysis task. Simultaneously, the attention mechanism is applied at the Bi-RNN output layers to pay suitable attention to different documents. We selected SD4A, Sentiment140, and US-Airline Twitter datasets for short sentences because they are challenging due to many misspellings, including polysemy and informal language, and IDMB for the long sentence to prove that the model is suitable for short and long intro sentence sentiment. Social media users commonly post abbreviations and misspellings, so one spelling mistake may change the whole sentence’s viewpoint. Likewise, we apply a unique data preprocessing task that removes noise by performing spell correction, word segmentation, and tokenization to address this problem. We experimented on a diverse number of accessible benchmark datasets to test our proposed model’s effectiveness. The significant contribution of this article is as follows:

- This work proposed a novel deep model for sentiment analysis. The model adopts the advantages of local feature extraction by CNN and characteristics of BiLSTM and BiGRU, considering contextual information that effectively improves sentiment knowledge and accuracy.
- We offer data preprocessing to acquire structured data for remarkable outcomes. This process corrects the spelling mistakes, removes the noise, performs tokenization, word lemmatization, and normalization techniques.
- The attention mechanism is used to pay suitable attention to different words to improve the feature expression ability, and Gaussian Noise is applied to avoid overfitting.
- We performed a comparative experiment on four public datasets to assess the proposed architecture’s effectiveness by improving long and short sentences.

The remaining of the article is categorized as follows. “Related Work” explains the related work. “Proposed Architecture” describes the architecture of the framework. “Experiments” provides the experiment setup and implementation, and “Results Analysis and Discussion” presents the results analysis and discussion. Finally, “Conclusion” elaborates the conclusion and futures work.

## Related Work

With a large amount of user-generated text, SA becomes an important part of NLP Abdi et al. (2019). SA is the text classification process into different categories (*e.g*., positive or negative, etc.). SA can be categorized into three levels (Hussein, 2018; Yessenalina, Yue & Cardie, 2010) sentence-level (Farra et al., 2010), document level (Yessenalina, Yue & Cardie, 2010), aspect-based level (Hu & Liu, 2004; Wang et al., 2016b). Sentence-level SA aims to determine whether the opinions expressed in sentences are positive, negative, or neutral (Liu et al., 2014). The document-level SA is a text processing technique to the distinguish sentiment polarities of the overall given document. It will be considering the document expresses only one single topic. Supervised traditional machine learning classifiers such as NB, SVM, and maximum entropy (ME) are used for document-level sentiment classification on different features. For example, semantic features of movie review (Kennedy & Inkpen, 2006), unigram, bigram, POS-tags, position information (Pang, Lee & Vaithyanathan, 2002), and discourse features opinion polarity classification have been proposed (Somasundaran et al., 2009). Most sentiment analysis techniques follow three methods (Sun, Luo & Chen, 2017) lexicon-based, machine learning, and hybrid approach sentiment analysis. These methods are expendable and straightforward. However, they have severe limitations, such as being dependent on human effort labeling, long-term activity, and limited effectiveness leading to a conversational and unstructured social networks text (Saeed, Ayaz Abbasi & Razzak, 2020; Saeed et al., 2019). To address domain dependency problem, Ghiassi & Lee (2018) proposed an approach based on a transferable domain lexicon and supervised ML for Twitter data. In this method, dynamic architecture for neural networks (DAN2) and SVM are used as a “one *vs*. all” approach for sentiment classification. Two ML tools were utilized to avoid domain dependency. The method achieved accurate results and reduced the feature set to a small set of seven “meta-features” to reduce sparsity. Recently, researchers used deep learning and achieved remarkable success in NLP and computer vision tasks such as network traffic analysis (Woźniak et al., 2021a). Woźniak et al. (2021b) developed a system to detect body movement using RNN and sensor reading. The results evaluation of these methods shows outperforming performance with an accuracy of 99%.

### Deep models for sentiment analysis

Most techniques proposed for sentiment classification through DL are geared towards semantic modeling. CNN is generally known as a feed-forward neural network (Ju et al., 2019) to formulate a mechanism of multiple filters by considering layer-to-layer convolution that enables it to extract the significant hidden features from the datasets. It considers the relationship between global information and can extract these features (Liao et al., 2017).

Although numerous neural networks have been widely studied for feature selections (Alayba et al., 2018; Kumar, Verma & Sharan, 2020). Chen et al. (2019) suggested conducting further robust research using multiple neural networks to acquire better achievements. Rezaeinia et al. (2019) proposed Improved word vectors (IWV) to increase the accuracy of pre-trained word embedding. This method combined GloVe, Part-of-Speech (POS), and lexicon-based approaches and then tested *via* three CNN layers. Other variants of the CNN network for sentiment analysis applications include charCNN (Dos Santos & Gatti, 2014), Glove-DCNN (Jianqiang, Xiaolin & Xuejun, 2018), CRNN (Wang et al., 2016a), DNN (Dang, Moreno-García & De la Prieta, 2020), and many more. However, these models face vanishing gradient problems and did not consider long dependencies. The current study proposes a new DL model for polarity detection based on the RNN and its variant’s ability to overcome this problem. Nguyen & Nguyen (2019) proposed a method based on CNN and RNN to capture local dependencies and memorize long-distance information. The authors integrated the advantage of various DL models to reduce overfitting in training. However, this model accuracy is not very significant for larger datasets. Zang et al. (2020), utilizing CNN and LSTM hybrid models to detect multidimensional features and time-series attributes, succeeded in attaining better results than before and provided an idea to further correlation models. This idea had been applied by Yoon & Kim (2017) when they proposed a novel SA framework by a new correlation of CNN and BiLSTM in a statistical manner.

Similarly, Wang, Jiang & Luo (2016) combined the CNN and RNN for SA, wherein the LSTM and CNN layers have been structured into numeral order. The results outperformed the Stanford Sentiment Treebank dataset with the provided model. Habimana et al. (2020) presented a novel technique by considering the contextual features of the sentiment classification using a convolutional gated recurrent network (ACGRN) to prioritize the feature knowledge for improved sentiment detection. The experiments were conducted at six different size real-time datasets and found that the proposed approach outperforms significantly. However, the author suggested examining the proposed model for sequence text, which probably comprises long-term dependencies. An enriched form of RNN is LSTM, capable of automatically adding or removing the progressive state information automatically (Liu, Mi & Li, 2018). LSTM enables the models to control the gradient vanishing during the long-term dealings about feature extraction of compound time-series and sequences. Kumar, Verma & Sharan (2020) executes an analytical study to solve aspect-based SA using the DL neural network (ATE-SPD) for sentiment polarity detection. Their proposed model solved that issue by considering the polarities of online reviews after sequence labeling. It used BiLSTM to combine a conditional random field (CRF) approach and is regarded as the best sequence labeling approach. It confirmed that CRF enabled BiLSTM to improve the efficiency of the proposed model. Recently, numerous sentiment resources and linguistic information are being used in sentiment classification. SA tasks are perceived as sequential tasks that rely on a particular vector and cause text information loss if the vector length is too short. LSTM has been comprehensively studied in Alayba et al. (2018), Wang et al. (2016a), Chen & Wang, 2018, Kaladevi & Thyagarajah (2019), Wang et al. (2020), Alqushaibi et al. (2021) for the extraordinary outputs of sentiment polarity classification with the combination of other neural networks, *e.g*., CNN. However, the LSTM and CNN combination efficiency relies on the quantity and quality of datasets.

Jin et al. (2020) proposed a multi-model named Multi-Task Learning Multi-Task Scale with CNN and LSTM (MTL-MSCNN-LSTM) for sentiment classification of multi-tasking environment. According to the author, the proposed model appropriately managed the vast amount of local and global features with various text scales to signify the sentiment’s accuracy with F1 scores. However, the efficiency comparison with single-task learning was not worthy because multiclass sentiment utilizes the NLP process. Li et al. (2020b) proposed another method by combining the CNN-BiLSTM models to improve the sentiment knowledge by sentiment padding for each comment and review. It took advantage of the sentiment lexicon and integrated a parallel CNN and BiLSTM to enhance the polarity’s sentiment information. Chatterjee et al. (2019) propose a deep Learning-based approach for emotion detection in textual dialogues called SS-BED. The SS-BED uses two-word embeddings matrices, and the output is fed into two LSTM layers to extract sentiment and semantic for emotion recognition. However, besides the specific advantages and disadvantages, a general weakness of those models is that they cannot consider each sentence’s importance differently. Recently researchers have focused on attention mechanisms to solve this issue.

### Attention-based models

Li et al. (2019) proposed a DNN approach *via* an attention mechanism for text sentiment classification. This model integrated sentiment linguistic knowledge into the DNN to learn sentiment feature enhanced word representation and decrease the gap between sentiment linguistic knowledge and the DL methods. The CA-LSTM model (Feng et al., 2019) incorporates preceding tweets with word-level and tweet attention for context-aware microblog sentiment classification. However, these models decode the input file into a specific length vector. If the length of the defined vector is set shorter or longer, the information will be lost. To solve this issue, Li et al. (2020a) proposed a multichannel BiLSTM model with self-attention mechanism (SAMF-BiLSTM). This model uses multichannel BiLSTM and the outputs fed into the self-attention mechanism to enhance the sentiment information. However, the author stated that this model needs to redesign the mechanism for a particular document-level classification, and the author did not find the solution for long text. CNN attention-based and BilSTM (AC-BiLSTM) (Liu & Guo, 2019) proposed to address the high dimensionality and sparsity of text data on natural language processing. In this method, CNN is used to extract the high-level phrase, BiLSTM is applied to access forward-backward context presentation. Attention mechanism employed at the output of BilSTM to give different focus on the features. Even though their work results are encouraging in terms of classification accuracy, this method did not consider overfitting problems, and the author stated that this model needs improvement and redesign. AttDR-2DCNN model (Liu et al., 2020) helps combat the pending task of long text for document classification and discover the dependencies among features. While findings of AttDR-2DCNN lacks value about words in a sentence to enhance the polarity classification. Basiri et al. (2021) proposed a model that used two LSTM and GRU layers to extract the context, and the attention mechanism is applied to emphasize different words. The authors use multiple directions to affect the model’s accuracy. However, neither method achieved satisfactory results for large datasets, such as sentiment140. In our previous study, attention-based CNN and BiLSTM Model for Sentiment Analysis (Kamyab, Liu & Adjeisah, 2021). CNN is employed to learn high-level feature context and, the attention mechanism is applied at the CNN output layers to pay suitable attention to different documents. BiLSTM extracts contextual information from the feature generated from previous layers. This model achieved significant accuracy on large datasets for short text. However, it still needs improvements. Our proposed model address the issues and weaknesses mentioned earlier, leveraging CNN, BiLSTM, and BiGRU.

## Proposed Architecture

**Figure 1:**
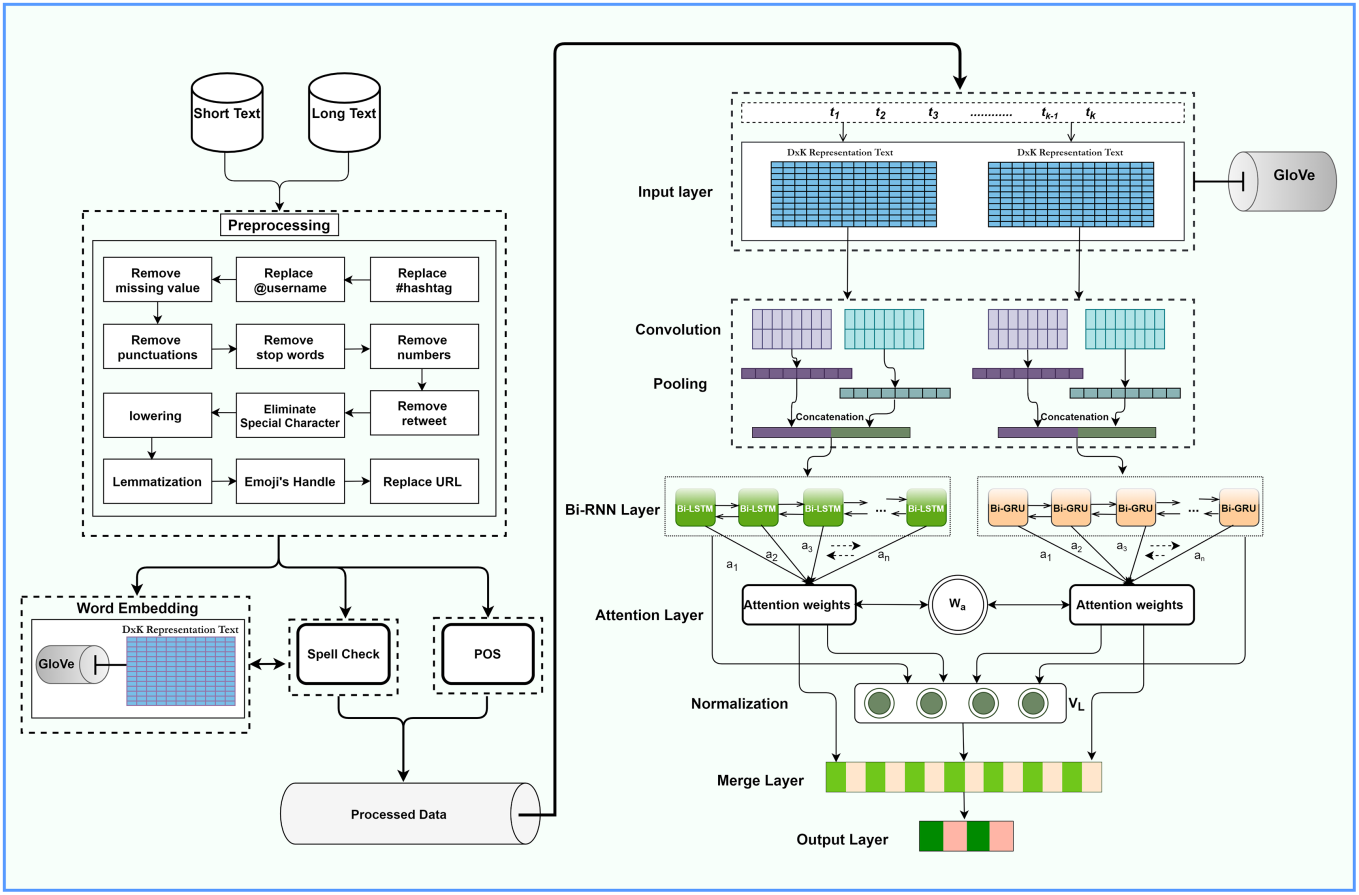
The overall architecture of the ACR-SA model.

Our proposed architecture is illustrated in Fig. 1. It comprises a distinctive data preprocessor, text representation layer, CNN and pooling layers, bidirectional RNN, an attention layer, fully-connected layer, and output layers. We apply word representation for spell check and noise removal during preprocessing. Glove word embedding represents the vector, while CNN extracts local features. Bi-RNN takes CNN feature-generated data as input and extracts the long dependencies feature as an output during training. The attention mechanism at the output layers of RNN and CNN pays suitable attention to different words. The following sections discuss each layer in detail.

### Data pre-processor

The language used on social media is non-standard, informal, with many grammatical errors, acronyms, misspellings, and non-standard punctuation. Therefore, they are in serious need of extensive processing. We designed a unique data preprocessing step to control and deal with all issues. These methods involve various steps such as deleting non-Unicode strings, non-English characters, replacing URLs, and User Handler. We also did spelling correction, partial speech tagging (POS), and word formation. Table 1 shows an example of data preprocessing to Twitter data. We employed Glove with 300 dimensions for spell check to significantly correct all the misspelled words as it is trained on 840 million tokens from a standard crawl.

**Table 1:** Comparison of original and structured text given of the preprocessing datasets.

Unprepared text	Prepared text
@AmericanAir lovely flight back from MIA to LHR - great crew - thanks :-))	USER_MENTION lovely flight back from mia to lhr great crew thanks EMO_POSITIVE
@AmericanAir I have emailed several tyms. hand wrote a letter, went to airport, called. please fix this! Or @abc7newsBayArea please help!	USER_MENTION i have emailed several times. hand wrote a letter went to airport called. please fix this or USER_MENTION please help
p.s. I didn’t realize that you were sending me messages	ps i did not realize that you were sending me messages

### Input layer

As stated, we used Glove to represent words using similarities in words. The input layer receives text data as *T*(*t*_1_, *t*_2_,…, *t*_*k*_), where *t*_1_, *t*_2_,…, *t*_*k*_ are the *k* number of token with dimensions of each input word *d*. Hence, *R*^*d*^ will be the dimensions space of each word or token; then each word is converted into word vector of *d* dimension, the word vector matrix *T* corresponding to a sentence of length *k* can be obtained as:

(1) $$T = \{ {t_1},{t_2},{\rm }...,{t_k}\} \in {R^{k \times d}}$$

We apply the zero-padding strategy since the input text length is not the same. If the length is longer than the predefined length, then uniform(*l*) will be truncated, but if the length is shorter than the predefined length, then *l* zero padding will be added to the size. The input text matrix generation after uniform length shows as Eq. (2):

(2) $$T \in {R^{l \times d}},$$

where, the input text *T* is passed to the Glove embedding layer to generate the word embedding vector from the *corpus* text. In addition, Gaussian Noise is applied after receiving text representation from the embedding layer. The Gaussian Noise *z* ∼ *N*(0, *σ*^2^) process can be used as a regularization method, making the model more vital and less prone to overfitting. Since Gaussian noise is applied directly to the word embedding, this process serves as a random data augmentation during training time.

### Convolutional neural network

Two-word representations are then fed to the two convolutional layers based on row representation. Let’s assume the *i*th row input feature matrix corresponding to a single channel is *V*_*i*_ ∈ *R*^*d*^, where *V*_*i*_ is the feature vector of *i*th word with *d* dimensional. *V*_*i*:*i*+*l*−1_ ∈ *R*^*l*^ ^*d^ represent the feature matrix composite of *l* input text length from *i*th word to the *i* + *l* − 1 word. Then feature *h*_*i*_ after extraction is formulated as follows:

(3) $${h_i} = f(W * {V_{i:i + l - 1}} + b + z),$$

where *f* is the activation function for the non-linearity, *h*_*i*_ represents *i*th feature value obtained by the convolutional operation of each sentence. *W* represents the matrix’s weight, *b* is the bias, *z* Gaussian noise. We have two Chanel; *h*_1:*i*_ and *h*_2:*i*_ get two outputs from the convolutional layers in each channel. The feature obtained from the CNN layers are stated as follows:

(4) $${h_n} = [{h_1},{h_2},...,{h_i}],n = 1$$

(5) $${h_n} = [{h_1},{h_2},...,{h_i}],n = 2$$

Next, is to apply the max-pooling to each channel output *h*_*n*_ layer to minimize the dimensions of the dataset as shown in Eqs. (6) and (7).

(6) $${p_n} = Max[{h_n}],n = 1,$$

(7) $${p_n} = Max[{h_n}],n = 2,$$

where the *P*_*n*_, *n* = 1 is the feature map obtained after the max-pooling layer from the first convolutional kernel, and *P*_*n*_, *n* = 2 feature map obtained after the max-pooling layer from the second convolutional kernel.

### RNN layer

RNN is a dominant model due to the compound configuration for NLP tasks of feature extraction. Studies show that RNN is has been applied successfully for sequential data. It can utilize its internal memory to execute variable sequences of datasets, making it applicable to investigate sentiment polarity (Hochreiter & Schmidhuber, 1997). To solve the vanishing gradient problem, the previous steps out transfer into two independent bidirectional RNN.

#### Long short-term memory

LSTM is an enhanced RNN that can handle the vanishing problems of RNN. LSTM is proposed as a gating mechanism that comprises three gates (input, forgets, and output). The adaptive gating mechanism generates output from the current states, remembers and sends it to the next stage as input every time. LSTM comprises the memory cell *C*_*t*_ that considers the time interval of its state over arbitrary. It consists of three different non-linear gates; Input gate *i*_*t*_, Output gate *O*_*t*_, and Forget gate *f*_*t*_, responsible for information flow to and from the *C*_*t*_. Equations (8)–(13) (Socher et al., 2013) presents the function of LSTM gates. Wherein, *σ* (.) is a sigmoid function based on element, tanh(·) is a hyperbolic Tangent function, and ${\rm \odot }$ is the product. Similarly, *X*_*t*_ is the input vector while *h*_*t*_ is the hidden vector for a time *t*. Meanwhile, *U* and *W* offering the weight matrix of input and hidden vector, and *b* stands for bias vector (Liu & Guo, 2019; Alom, Carminati & Ferrari, 2020).

(8) $${f_t} = \sigma ({W_f}{h_{t - 1}} + {U_f}{x_t} + {b_f})$$

The forget gate (*f*_*t*_) is liable for ignoring or forgetting information.

(9) $${i_t} = \sigma ({W_i}{h_{t - 1}} + {U_i}{x_t} + {b_i})$$

(10) $${u_t} = \tanh ({W_c}{h_{t - 1}} + {U_c}{x_t} + {b_c})$$

(11) $${c_t} = {f_t}{\rm \odot }{c_{t - 1}} + {i_t}{\rm \odot }{u_t}$$

The input gate (*i*_*t*_) decides what information should store in the memory cell according to Eqs. (9)–(11). While in the LSTM network, forget gate decides about the output information, the amount of information, and which parts of the cell state should be output according to the following Eqs. (12) and (13).

(12) $${o_t} = \sigma ({W_o}{h_{t - 1}} + {U_o}{x_t} + {b_o})$$

(13) $${h_t} = {o_t}{\rm \odot }\tanh ({c_t})$$

LSTM network these gates unit helps to remember important information over multiple time updates and ignore the information which is not required.

#### Gated recurrent unit

GRU is a similar variant of LSTM, but has only two gates, an update gates (*z*_*t*_) and the reset gates (*r*_*t*_). These two gates control the information updating process to the state together. Reset gate *r*_*t*_ controls the contributions of the past state *h*_*t*−1_ to the candidate state ${\tilde h_t}$ and the smaller the value is, the higher the ignored rate. At the time *t*, the reset gate works as follows.

(14) $${r_t} = \sigma ({W_r}{h_{t - 1}} + {U_r}{x_t} + {b_r})$$

The update *z*_*t*_ is used to determine how much of the stream information can be kept or forgotten at the timestamps of *t* and *t* − 1, the update gate expressed as follows.

(15) $${z_t} = \sigma ({W_z}{h_{t - 1}} + {U_z}{x_t} + {b_z}),$$

where *σ* is the logistic sigmoid function, *x*_*t*_ and *h*_*t*−1_ respectively denote the input and previous hidden states. The GRU state at the time interval *t* is calculated as Eq. (16), and the candidate state ${\tilde h_t}$ is computed as Eq. (17).

(16) $${h_t} = (1 - {z_t}){\rm \odot }{\tilde h_t} + {z_t}{\rm \odot } + {h_{t - 1}}$$

(17) $${\tilde h_t} = \tanh ({W_h}({h_{t - 1}}{\rm \odot }{r_t}) + {U_h}{x_t} + {b_h}),$$

like LSTM, *W*, *U* are learnable weights, and *b* is the bias term where ⊙ is vector element multiplication.

#### Bidirectional RNN

This paper used two independent bidirectional RNN (LSTM and GRU) to consider both forward and backward features in parallel. The purpose of using two different RNN is to get better results in both large and small datasets. BiGRU is applied to get richer context information, and GRU bit faster or needs less data to generalize. However, BiLSTM generates final features by sequentially processing the map, leading to better results with large data. These two independent Bi-RNN networks are applied to form the feature context matrix representation from the vectors obtained from two channels of the previous steps with *m* padding length or maximum feature-length.

(18) $${\overrightarrow h _{{f_{lstm}}}} = \overrightarrow {LSTM} ({p_n}),n \in [1,m]$$$${\vec{h}}_{f_{lstm}}=\vec{LSTM}(p_{n}),n\in [1,m]$$

(19) $${\overleftarrow h _{{b_{lstm}}}} = \overleftarrow {LSTM} ({p_n}),n \in [m,1]$$

Equation (20) represent forward, and (21) represent backward GRUs.

(20) $${\overrightarrow h _{{f_{gru}}}} = \overrightarrow {GRU} ({p_n}),n \in [1,m]$$

(21) $${\overleftarrow h _{{b_{gru}}}} = \overleftarrow {GRU} ({p_n}),n \in [m,1]$$

We now obtain an annotation for each input vector by concatenating the forward and backward context in Eqs. (22) and (23).

(22) $${h_{{t_{lstm}}}} = LSTM[{\overrightarrow h _{{f_{lstm}}}},{\overleftarrow h _{{b_{lstm}}}}]$$

(23) $${h_{{t_{gru}}}} = GRU[{\overrightarrow h _{{f_{gru}}}},{\overleftarrow h _{{b_{gru}}}}],$$

where, *h_t_*_*lstm*_ is the concatenating output of forwarding $({\overrightarrow h _{{f_{lstm}}}})$ and backward $({\overleftarrow h _{{b_{lstm}}}})$ extracts long dependencies feature from BiLSTM. Similarly, *h_t_*_*gru*_ is the concatenating output of forwarding $({\overrightarrow h _{{f_{gru}}}})$ and backward $({\overleftarrow h _{{b_{gru}}}})$ extracts long dependencies feature from BiGRU. The extracted feature of the entire sentence is $[{\overrightarrow h _{{f_{lstm}}}},{\overleftarrow h _{{b_{lstm}}}}]$ and $[{\overrightarrow h _{{f_{gru}}}},{\overleftarrow h _{{b_{gru}}}}]$. In this way, the forward and backward contexts can be considered simultaneously.

### Attention mechanism

Since different words have different meanings, we apply attention to each Bi-RNN generated feature to emphasize the meaning of each sentence. The *h_t_*_*lstm*_ and *h_t_*_*gru*_ word annotation each one is passed through one layer perceptron to get *u_t_*_*gru*_ as the hidden representation of *h_t_*_*lstm*_ for *t*th input and *u_t_*_*gru*_ as the hidden representation of *h_t_*_*gru*_ *t*th input that formulated as follows:

(24) $${u_{{t_{lstm}}}} = \tanh (w * {h_{{t_{lstm}}}} + b)$$

(25) $${u_{{t_{gru}}}} = \tanh (w * {h_{{t_{gru}}}} + b),$$

where *w* represent the weight and *b* is bias, then to measure the importance of words, we perform normalization on the hidden representation of *u*_*t*_ with context vector *v*_*s*_ which is randomly initialized and jointly learned during the training process presented as:

(26) $${A_{{t_{lstm}}}} = \displaystyle{{exp({u_{{t_{lstm}}}} * {v_s})} \over {\sum_{i = 1}^m exp({u_{{t_{lstm}}}} * {v_s})}}$$

(27) $${A_{{t_{gru}}}} = \displaystyle{{exp({u_{{t_{gru}}}} * {v_s})} \over {\sum_{i = 1}^m exp({u_{{t_{gru}}}} * {v_s})}},$$

in Eqs. (26) and (27), *A_t_*_*lstm*_ and *A_t_*_*gru*_ represent the normalized weight LSTM and GRU layers respectively, *m* the number of words in the text, and exp(.) is the exponential function. Then we concatenate the LSTM and GRU achieved normalized weight *A*_*t*_, and it can be expressed as Eq. (28). These importance weights *A*_*t*_ are aggregated into *V* to obtain a single vector can be denoted as Eq. (29).

(28) $${A_t} = {A_{{t_{lstm}}}} \oplus {A_{{t_{gru}}}}$$

(29) $$V = \sum ({A_t} * {h_t})$$

### Output layer

This layer performs the significant sentiment classification task by utilizing the merge feature layer, as illustrated in Fig. 1. The Sigmoid function is considered for binary input, while cross-entropy is responsible for the discrepancy between the actual and predicted sentiments.

## Experiments

This section discusses the data description, hyper-parameters setting, and baseline. The proposed model was implemented using data preprocessing, word representation, and joint deep learning networks with CNN and Bi-RNN. We test the ACR-SA model on SD4A, sentiment140, US_airline datasets as short text, and IMDB as a long text dataset considering the ultimate goal of accurately analyzing our proposed model. We conducted several experiments to evaluate the performance of our architecture. The model was trained on 50 epochs and compared our results with previous models.

### Datasets

This study has considered SD4A, Sentiment140, US-Airline for short text, and IMDB for long text. The acquisition of SD4A is also a significant contribution to this work. The rest of the three datasets are the existing SOTA datasets in various studies with substantial performance for the other models. Detailed statistics of the dataset are listed in Table 2.

**Table 2:** Detailed statistics of the dataset used to verify our model’s effectiveness.

Dataset	Positive	Negative	Total
SD4A	18,309	18,539	36,848
Sentiment140	248,576	80,000	1,048,576
US Airline	2,343	9,112	11,455
IMDB	25,000	25,000	50,000

#### Sentiment dataset for Afghanistan (SD4A)

We collected this dataset about Afghanistan’s (https://www.kaggle.com/kamyab20/sentiment-dataset-for-afghanistan-sd4a) security status from 29/03/2018 to 21/06/2018. The dataset includes 56 k tweets with positive, negative, and neutral classification (Kamyab et al., 2018; Kamyab, Liu & Adjeisah, 2021). However, we consider only positive and negative tweets from this dataset, given that this work explores binary classification problems. After removing the neutral and duplicate tweets, we got 36,848 tweets containing 18,309 positive and 18,539 negative tweets, respectively.

#### Sentiment140

The dataset is scraped from Twitter by Sandford (http://help.sentiment140.com/for-students) graduate students (66). Currently, the sentiment140 dataset is one of the mostly used and standard datasets for text classification, with 1,048,576 tweets automatically categorized into 248,576 positive and 80,000 negatives. It has been employed in various SOTA studies (Song et al., 2020; Rezaeinia et al., 2019; Dos Santos & Gatti, 2014; Wang et al., 2016a; Dang, Moreno-García & De la Prieta, 2020; Nguyen & Nguyen, 2019; Basiri et al., 2021; Alec & Richa Bhayani, 2009; Kamyab, Liu & Adjeisah, 2021).

#### US-Airline

US Airline dataset is available on the Kaggle (https://www.kaggle.com/crowdflower/twitter-airline-sentiment) website, which contains 14,641 tweets. This dataset crawled in 2015 about the US-Airline problems. In this research, we consider only positive and negative tweets. After removing neural tweets, we obtain 11,541 tweets for this research as it has been adopted in benchmark literature (Jianqiang, Xiaolin & Xuejun, 2018; Chatterjee et al., 2019; Liu & Guo, 2019; Socher et al., 2013; Kamyab, Liu & Adjeisah, 2021).

#### IMDB

IMDB dataset is available on the Kaggle (https://www.kaggle.com/lakshmi25npathi/imdb-dataset-of-50k-movie-reviews) website is includes 50,000 reviews with polarities of 25,000 positives and 25,000 negatives. The existing research work (Liu & Guo, 2019; Rezaeinia et al., 2019; Dang, Moreno-García & De la Prieta, 2020; Basiri et al., 2021; Rasool et al., 2021) has also utilized this benchmark dataset.

### Training procedure and hyper-parameters setting

The proposed model and selected baselines are implemented based on the *Keras* deep learning API written in python language. The models are executed on the Windows operating system with 3.00 GHz processor and 16 GB RAM. We used the grid search optimization techniques to find the hyperparameters used in the experiments. The hyper-parameters values have used Glove with 200 dimensions for word embedding. The input layer matrix T, 90,000 words vocabulary size applied to the dataset. In addition to traditional preprocessing, we used the *multiprocessing* API *text*_*similarity*_*spellings* function to predict a word in a sentence based on the context and correct the spell mistakes. In training, 80% of the observations were used for training, 10% for validation, and 10% for testing, and the 200 and 40 first words were considered in long and short text using padding process, respectively. Gaussian Noise with the value of 0.3 and Dropout with the value of 0.4 applied at the connection network with the two-channel convolutional layer to avoid overfitting. We used 64 bias with kernel sizes of 4 and ReLU as an activation function in the convolutional layers. The output of the convolutional layer fits into max-pooling with pool size 2. Two independent Bi-RNN (LSTM and GRU) were applied with 256 sizes, dense size 128, and ReLU as the activation function. Attention mechanism applied after received feature from each bidirectional RNN layer, and the output sent to the concatenation layer. After the dense layer, a Sigmoid function is used for the binary classifier. Finally, binary cross-entropy is used for the training model, and Adam optimizes the model’s learning rate. Table 3 describes the hyper-parameters value in our model.

**Table 3:** Hyper-parameters setting for proposed model training.

Hyperparameter	Value
Number of epochs	[1–50]
Batch size	128
Embedding dimension	200
Embedding padding size	[40–200]
GaussianNoise rate	0.3
GaussianDropout rate	0.4
Number of CNN layers	4
Window size *l* of Convolutional kernel	[2,3,2,3]
Convolutional kernel bias	64
Number of Pooling	4
Pooling size	2
BiGRU layer output size	[49,256]
BiLSTM layer output size	[49,256]
Number of Dropout	2
Dropout rate	0.2
Number of batch normalization	2
Number of attention mechanism A	2
Attention with context	w = (256 × 256), *b* = 256, *u* = 245
Number of dense layer	3
Learning rate	0.0001
Optimizer	Adam
Activation function type	ReLu, tanh, Sigmoid

### Baseline methods

In this model, four datasets are used, and we used several recent similar models developed for sentiment classification. The list of the most current and significant baseline models that we compared our model with are as follows.

- CRNN (Wang et al., 2016a): In the CRNN model, each sentence is considered a region, and a regional CNN is applied to the input word vectors. Max-pooling is used to reduce the local features’ dimensionality. Lastly, the LSTM layer is applied to capture long dependencies.
- SS-BED (Chatterjee et al., 2019): In this model, two-word embedding matrices are used for word representation, then two LSTM layers are applied for learning the semantic and sentiment knowledge. And finally, fully connected layer and hidden layer to classify the emotion categories.
- ARC (Wen & Li, 2018): A well-known model for word representation, which takes input to the RNN layer that fed the results into the CNN layer for attention mechanism implication.
- ABCDM (Basiri et al., 2021): This is an attention-based bidirectional CNN-RNN model. The model is combined from two bidirectional independent RNN layers for extracting the past and future features. The CNN layer’s applied to the output RNNs layer to reduce the dimensionality.
- Improved Word Vectors (IWV) (Rezaeinia et al., 2019): This model integrated three CNN layers, one max-pooling layer, and a fully connected layer for sentiment analysis. The authors combined Part-of-Speech (POS), lexicon-based approaches, and Word2Vec methods for vector improvement.
- AC-BiLSTM (Liu & Guo, 2019): This model starts with the CNN layer with different filter sizes for higher-level phrase extraction. The output of the CNN layer is fed into BiLSTM to access the preceding and succeeding context terms, followed by an attention mechanism.
- Word embedding-CNN (Alharbi & de Doncker, 2019; Dang, Moreno-García & De la Prieta, 2020): In this study, the authors propose the architecture of CNN that takes into account and user behavior. This model consists of five parts: input, convolution, pooling, activation, and softmax layers.
- Glove-RNN-CNN (Abid et al., 2019): This method was proposed for the Twitter datasets by merging RNN variants and CNN. The GloV pre-trained word embedding was employed as input and then fed the input into RNN layers after afterward connected with CNN. Although this method has yielded significant results for small datasets, it is not suitable for large datasets and long text.
- ACL (Kamyab, Liu & Adjeisah, 2021): In this model, CNN captured contextual features, and BiLSTM extracted long-term dependencies. Attention mechanism employed at the output of CNN to give different focus on the features. The GloV pre-trained word embedding was used for word representation.

## Results Analysis and Discussion

In this section, we provide the results of our model with the baseline methods. The experiment was executed on four datasets to evaluate the model accuracy, recall, precision, and F1 value’s effect. Besides, we visualize the impact of the attention mechanism on the words weight matrix and the effect normalization layer for decreasing overfitting. The experiment result validates that the proposed model (ACR-SA) performed better and achieved better accuracy than the other SOTA methods in all four datasets, as can be seen from Tables 4–7, in which the bolder entries indicate the highest performances of the models.

**Table 4:** Performance obtained on the Sentiment140 dataset.

Models	Class	Recall	Precision	F1	Accuracy
CRNN (Wang et al., 2016a)	N	0.9443	0.8682	0.9046	0.848
	P	0.5379	0.7497	0.6264	
SS-BED (Chatterjee et al., 2019)	N	0.9493	0.8807	0.9137	0.8632
	P	0.5856	0.7819	0.6697	
ARC (Wen & Li, 2018)	N	0.9468	0.8734	0.9086	0.8547
	P	0.5578	0.7648	0.6451	
ABCDM (Basiri et al., 2021)	N	0.9392	0.8945	0.9163	0.869
	P	0.643	0.7663	0.6993	
IWV (Rezaeinia et al., 2019)	N	0.9352	0.8942	0.9143	0.8661
	P	0.6434	0.755	0.6948	
AC-BiLSTM (Liu & Guo, 2019)	N	0.9412	0.878	0.9085	0.8552
	P	0.5784	0.7531	0.6543	
Word Embedding-CNN (Dang, Moreno-García & De la Prieta, 2020)	N	0.9448	0.8859	0.9144	0.865
	P	0.6078	0.7735	0.6807	
Glove-BiLSTM-CNN (Abid et al., 2019)	N	0.9391	0.8948	0.9164	0.8693
	P	0.6442	0.7665	0.7021	
Glove-BiGRU-CNN (Abid et al., 2019)	N	0.9416	0.8925	0.9164	0.8689
	P	0.6347	0.7713	0.6963	
ACL (Kamyab, Liu & Adjeisah, 2021)	N	0.9556	0.8842	0.9185	0.8706
	P	0.5968	0.8066	0.686	
ACR-SA	N	**0.9638**	**0.9098**	**0.936**	**0.9013**
	P	**0.6976**	**0.8587**	**0.7698**	

**Table 5:** Performance obtained on the US-Airline.

Models	Class	Recall	Precision	F1	Accuracy
CRNN (Wang et al., 2016a)	N	0.9429	0.9292	0.936	0.8965
	P	0.708	0.7532	0.7299	
SS-BED (Chatterjee et al., 2019)	N	0.9596	0.9233	0.9411	0.9036
	P	0.676	0.8048	0.7348	
ARC (Wen & Li, 2018)	N	0.9528	0.9398	0.9462	0.9031
	P	0.752	0.7966	0.7737	
ABCDM (Basiri et al., 2021)	N	0.9557	0.9662	0.9609	0.9376
	P	0.864	0.8276	0.8454	
IWV (Rezaeinia et al., 2019)	N	0.9616	0.9485	0.955	0.9273
	P	0.788	0.8347	0.8107	
AC-BiLSTM (Liu & Guo, 2019)	N	0.9724	0.9182	0.9446	0.9084
	P	0.648	0.8526	0.7364	
Word Embedding-CNN (Dang, Moreno-García & De la Prieta, 2020)	N	0.9606	0.9503	0.9555	0.9281
	P	0.796	0.8326	0.8139	
Glove-BiLSTM-CNN (Abid et al., 2019)	N	0.9646	0.9533	0.9589	0.9336
	P	0.808	0.8487	0.8379	
Glove-BiGRU-CNN (Abid et al., 2019)	N	0.9754	0.9438	0.9593	0.9336
	P	0.764	0.8843	0. 8197	
ACL (Kamyab, Liu & Adjeisah, 2021)	N	0.9671	0.953	0.9591	0.9401
	P	0.8478	0.8898	0.8683	
ACR-SA	N	**0.9947**	**0.9853**	**0.99**	**0.9842**
	P	**0.9455**	**0.9798**	**0.9624**	

**Table 6:** Performance obtained on the SD4A dataset.

Models	Class	Recall	Precision	F1	Accuracy
CRNN (Wang et al., 2016a)	N	0.8454	0.863	0.8541	0.8548
	P	0.8643	0.8468	0.8554	
SS-BED (Chatterjee et al., 2019)	N	0.8694	0.865	0.8672	0.8676
	P	0.8694	0.865	0.8672	
ARC (Wen & Li, 2018)	N	0.8851	0.8766	0.8808	0.8796
	P	0.874	0.8826	0.8783	
ABCDM (Basiri et al., 2021)	N	0.9309	0.9416	0.9362	0.9362
	P	0.9416	0.9309	0.9362	
IWV (Rezaeinia et al., 2019)	N	0.9162	0.9193	0.9178	0.9174
	P	0.9187	0.9155	0.9171	
AC-BiLSTM (Liu & Guo, 2019)	N	0.8737	0.8596	0.8666	0.8647
	P	0.8557	0.8701	0.8628	
Word Embedding-CNN (Dang, Moreno-García & De la Prieta, 2020)	N	0.9162	0.91	0.9131	0.9123
	P	0.9084	0.9146	0.9115	
Glove-BiLSTM-CNN (Abid et al., 2019)	N	0.9473	0.9399	0.9436	0.9431
	P	0.9387	0.9463	0.9425	
Glove-BiGRU-CNN (Abid et al., 2019)	N	0.9411	0.9492	0.9451	0.945
	P	0.949	0.9409	0.945	
ACL (Kamyab, Liu & Adjeisah, 2021)	N	0.9401	0.9471	0.9436	0.9453
	P	0.9503	0.9435	0.9469	
ACR-SA	N	**0.9506**	**0.955**	**0.9528**	**0.9539**
	P	**0.957**	**0.9528**	**0.9549**	

**Table 7:** Performance obtained on the IDBM dataset.

Models	Class	Recall	Precision	F1	Accuracy
CRNN (Wang et al., 2016a)	N	0.8397	0.8257	0.8327	0.8332
	P	0.8268	0.8408	0.8337	
SS-BED (Chatterjee et al., 2019)	N	0.8268	0.8397	0.8332	0.8364
	P	0.8458	0.8333	0.8395	
ARC (Wen & Li, 2018)	N	0.8571	0.854	0.8556	0.857
	P	0.8569	0.8599	0.8584	
ABCDM (Basiri et al., 2021)	N	0.8535	0.9024	0.8773	0.8842
	P	0.9098	0.8641	0.8864	
IWV (Rezaeinia et al., 2019)	N	**0.8851**	0.8392	0.8615	0.8594
	P	0.8343	0.8814	0.8572	
AC-BiLSTM (Liu & Guo, 2019)	N	0.8142	0.854	0.8336	0.8394
	P	0.864	0.8264	0.8448	
Word Embedding-CNN (Dang, Moreno-García & De la Prieta, 2020)	N	0.8422	0.8459	0.844	0.8462
	P	0.8501	0.8465	0.8483	
Glove-BiLSTM-CNN (Abid et al., 2019)	N	0.8531	0.8963	0.8741	0.8786
	P	0.9035	0.8629	0.8828	
Glove-BiGRU-CNN (Abid et al., 2019)	N	0.8834	0.876	0.8797	0.8708
	P	0.8778	0.8852	0.8815	
ACL (Kamyab, Liu & Adjeisah, 2021)	N	0.8489	0.884	0.8609	0.866
	P	0.8824	0.8501	0.8708	
ACR-SA	N	0.8597	**0.9336**	**0.8951**	**0.8998**
	P	**0.9395**	**0.8712**	**0.9041**	

Table 4, an overall glance for the Sentiment140 Twitter data shows that all these given models acquired 84.8% to 90.13% accuracies. Our proposed model achieved the highest accuracy of 90.13%. In contrast, the baseline highest accuracy 87.06% is achieved by ACL (Kamyab, Liu & Adjeisah, 2021). ACR-SA improvement achieved 2.02% F1, 1.66% precision, and 1.52% recall on the negative class and 7.01% F1, 5.21% precision, and 5.36% recall on the positive class than the baseline models.

Table 5 lists the obtained accuracies on different models on the US-Airline dataset. Comparing our proposed model with the baseline models, we can notice that our proposed model has a significant accuracy of 98.42% for this dataset. In contrast, ACL (Kamyab, Liu & Adjeisah, 2021) has the highest accuracy as compared to all other baseline methods. Meanwhile, it can be seen that the ACR-SA model improves F1, precision, and recall by 9.41%, 9%, and 8.15% in the positive class and 2.94%, 1.91%, and 1.93% in the negative class, respectively, than ABCDM (Basiri et al., 2021).

Table 6 presents the evaluation utilized for our proposed model on the SD4A dataset. The ACR-SA model gained 95.39% accuracy, which is sufficiently improved as compared to all baseline models. ACL (Kamyab, Liu & Adjeisah, 2021) and Glove-BiGRU-CNN (Abid et al., 2019) achieved the highest accuracy of 94.53%, 94.5% respectively among the selected baseline models, showing that our proposed model enhanced by 0.86%. Similarly, we can see that the ACR-SA model improves the F1, precision, and recall by 0.53%, 0.93%, 0.8% in the positive class, and 0.77%, 0.79%, 0.95% in the negative class, respectively, which is an efficient performance than highest values achieved in baselines.

Table 7 compares our proposed model with the selected baseline models on the IMDB dataset. All selected baseline models and our proposed model were tested. The ACR-SA model achieved 89.98%. Meanwhile, the ABCDM (Basiri et al., 2021) method achieved an accuracy of 88.40%, which is the highest among chosen baselines. Our proposed model was enhanced by 1.58% than the ABCDM model and 3.38% than the ACL model. Comparison of F1, precision, and recall shows that our model improves by 1.77%, 0.71%, and 2.97% respectively in the positive class, 1.78% F1, and 3.12% precision in the negative class. Only precision in the positive class did not improve.

To summarize the performance of ACR-SA compared to baselines, our proposed model outperformed accuracy and confusion matrices F1, precision, recall in all four datasets. In baseline methods, ABCDM (Basiri et al., 2021) performs well on the long-text issue among the baseline models. However, this model does not work significantly for short sentences because the first feature extraction layer is RNN, designed to capture long dependencies. Similarly, Glove-Bi-RNN-CNN (Abid et al., 2019) performs well on the small Twitter datasets, but this model does not use attention mechanism. In addition, ACL (Kamyab, Liu & Adjeisah, 2021) received sound performance for on short text. Nevertheless, the model did not address the co-occurrence of long dependencies. ACR-SA model solved this problem by using multiple layers to make it suitable for the big dataset. CNN was used as the first feature extraction of the model, which is suitable for short sentences. The zero-padding process and two independent bidirectional RNN make the model appropriate for long text.

**Figure 2:**
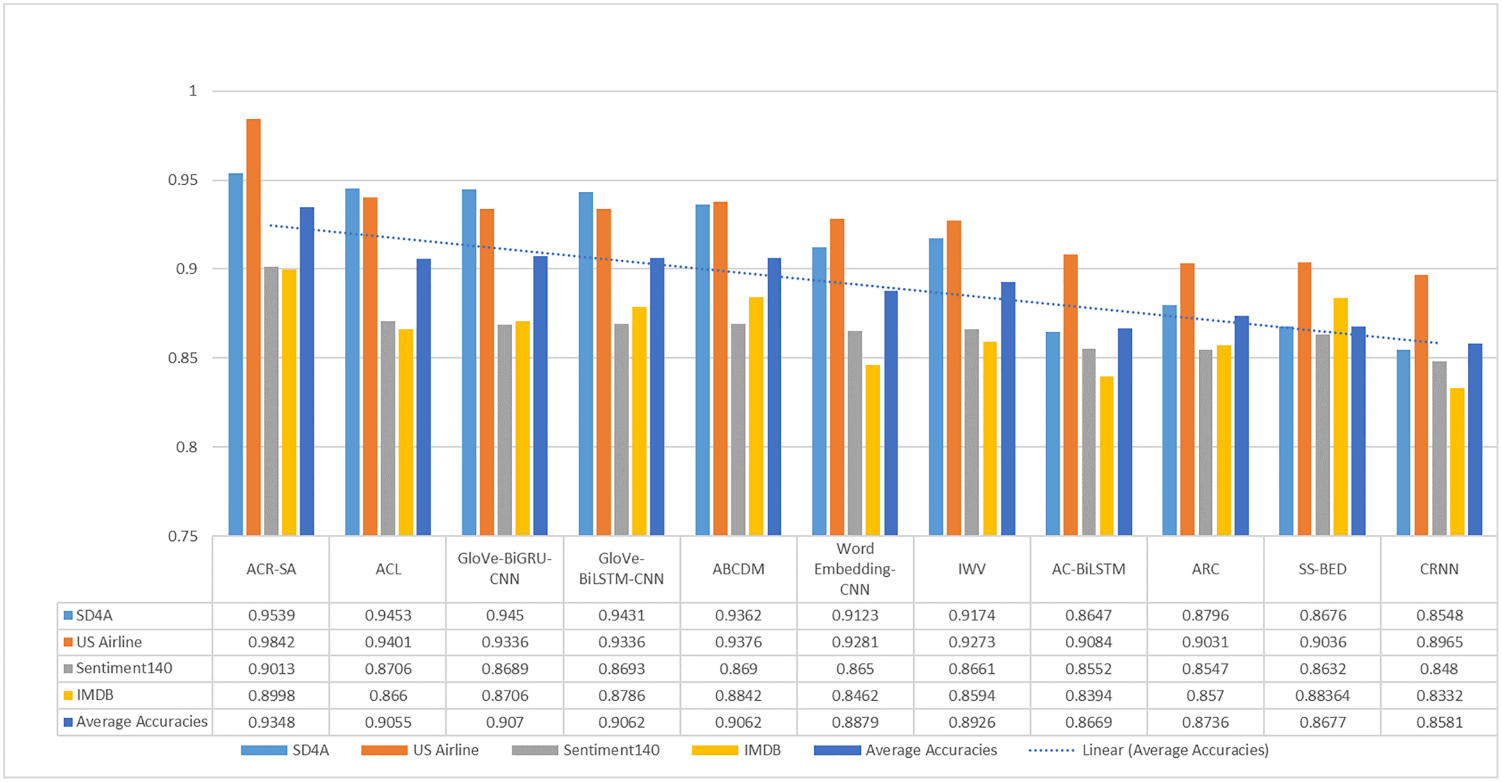
Comparative analysis of average accuracy of the baselines and proposed model on all given datasets.

For simplicity, Fig. 2 summarizes the proposed model’s accuracy with the aforementioned neural networks and datasets. It depicts that the proposed ACR-SA model successfully attained the highest accuracy with our structured dataset. In comparison with baselines, our proposed model achieved an excellent 4.41% accuracy in the US-Airline dataset, and 3.07% in the Sentiment140 dataset. Similarly, in SD4A and IMDB datasets the accuracies improved with 0.86% and 1.58% respectively, while the overall average accuracy improvement is 2.86%.

**Figure 3:**
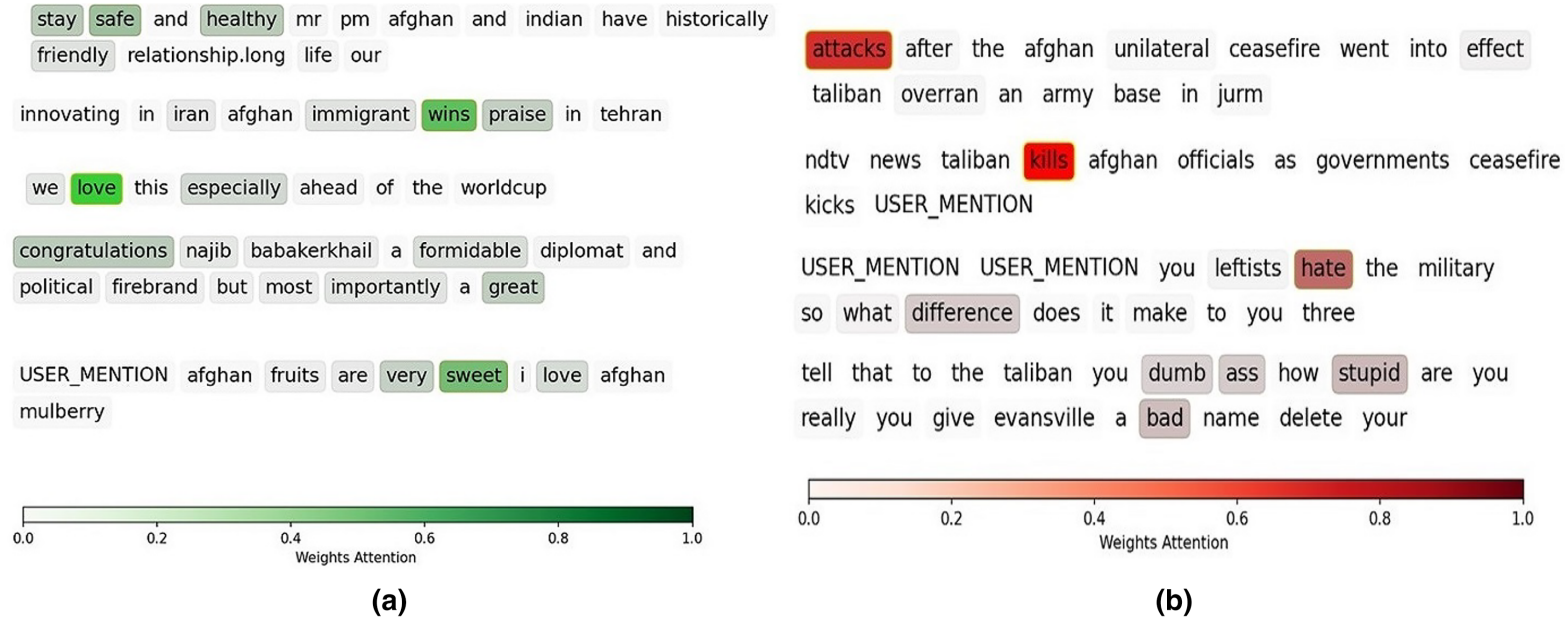
Illustration of attention mechanism on positive sentences (A), and negative sentences (B).

To evaluate the performance of the attention mechanism in our model, we select a few sentences for data visualization analysis, as shown in Figs. 3A and 3B. The darker the color, the more considerable weight of the given token. The depth of the color represents the corresponding word in the sentence. In Fig. 3A, the first sentence is positive. The importance weights of “stay,’’ “safe,’’ and “healthy’’ are larger than the other words on the same tweet. In Fig. 3B, the first tweet, the color of “attacks” is more profound than those of other words, indicates that it has more negative semantics than the other words.

**Figure 4:**
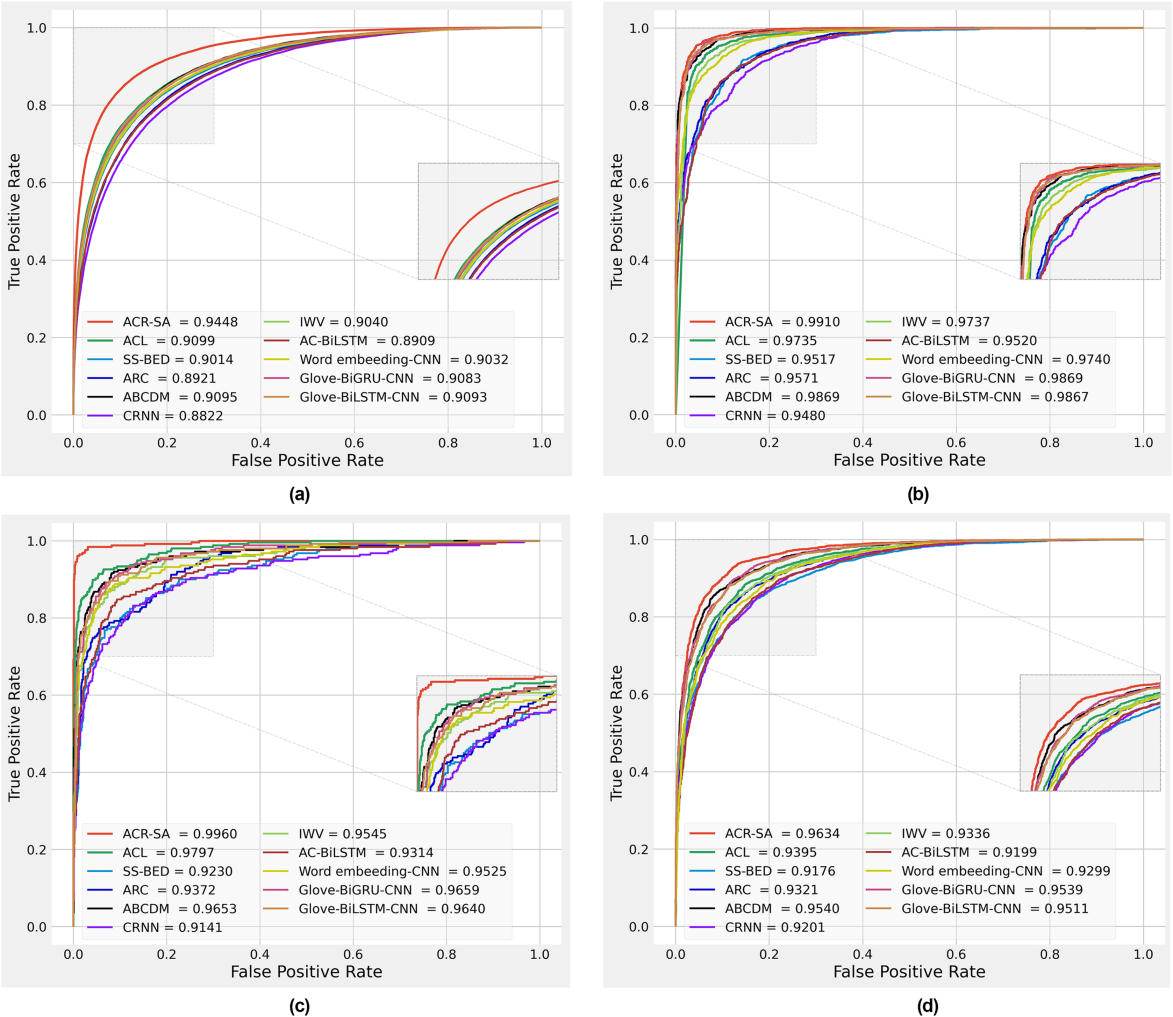
Performance comparison obtained by different methods on Sentiment140 dataset (A), SD4A dataset (B), US-Airline dataset (C), IMDB dataset (D).

We plotted the received operating characteristic curves of baseline models and the proposed model on the 10% test dataset, which is 10%. To interpret the prediction of models, as shown in Figs. 4A–4D. The area under the curve (AUC) is compared to evaluate the model’s performance. The results show that the proposed model performance is more significant than all baseline models. The improvements mainly benefit from preprocessing, two-channel CNN layers for the local feature extraction, two independent Bi-RNN by considering two directions of long dependencies, applying different normalization, and finally, weight attention according to the importance of each word.

**Figure 5:**
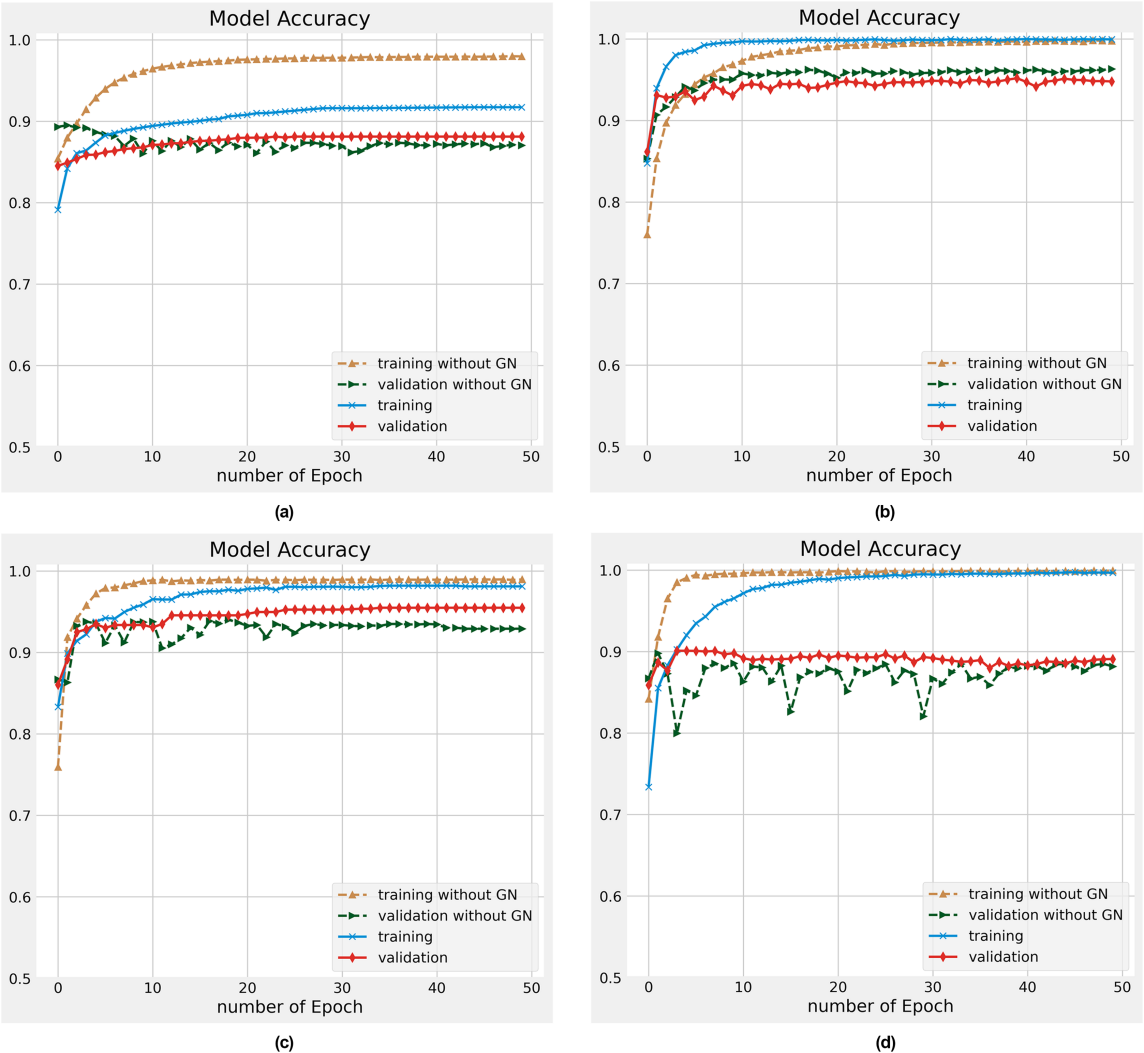
Accuracy performance on training and validation data on the (A) Sentiment140, (B) SD4A, (C) US-Airline, (D) IBDM datasets.

As discussed in the literature (Nguyen & Nguyen, 2019; Li et al., 2020a) DL models encounter overfitting challenges that reduce the model’s accuracy and performance. In this work, we resolve this challenging task by utilizing the Gaussian Noise (GN) on different datasets. The experiments are conducted on training and validation datasets with 50 epochs with and without Gaussian Noise. The individual outcomes of each data for training and validation data are demonstrated in Figs. 5A–5D. A glance at all these Figures, presenting the convergence for our proposed model that attained a reliable accuracy. We found that our proposed model significantly improved the overfitting problem in all different datasets. The training dataset’s accuracy is slightly higher after the number of 20 epochs than the validation or test dataset. While for initial epochs, the test data’s accuracy is a bit higher. Our proposed ACR-SA model converged toward the optimal solution after the mentioned number of epochs with consistent accuracy. These analyses provide evidence that the ACR-SA network reduces the overfitting problem and attains adequate accuracy.

## Conclusion

Deep learning models, especially CNN and RNN, are widely used for text classification. However, previous studies have the drawback of low accuracy and overfitting, which demanded to be tackled for significant knowledge extraction. We proposed a novel Attention-based deep model through two-channel CNN and Bi-RNN model for Sentiment Analysis (ACR-SA). The model combines unique data processing techniques, word representation, and DL techniques, including attention-based mechanisms. Data processing is applied to handle social media data challenges, such as spelling correction and harm model training accuracy. ACR-SA used pre-trained word embedding for word representation to create vector representation for each sentence. Further, different DL models were combined to extract higher features, capture long-term dependencies and generate sentiment analysis knowledge. Two-channel CNN layers were used to extract contextual features, to decrease the dimensionality of feature space, with max-pooling at the CNN layers’ output. Gaussian Noise and Dropout were used on the input layer to overcome the overfitting issues. The two independent bidirectional RNNs (LSTM and GRU) networks utilized to temporal feature and update the past and future sentiment representation of the CNN layers output. The attention mechanism was applied at the end of the BiLSTM and BiGRU layers to put more or less attention into different words. Moreover, this made the sentiment analysis more informative. Finally, a dense and fully connected layer with ReLU activation and sigmoid function transforms the vector into sentiment polarity classification. Experiments were conducted on three datasets of short-text English tweets and one dataset of long reviews about movies to analyze the performance of the proposed model for long and short text. Comparisons with SOTA baseline methods demonstrate that the ACR-SA is more effective and efficient in classifying and comprehending semantics in both short and long texts. Our proposed model’s performance achieved a magnitude of 95.39%, 98.42%, 90.13%, and 89.98% for the SD4A, US-Airline, Sentiment140, and IBDM datasets, respectively. In contrast, the overall average accuracy improvement is 2.86% compared to baseline methods results. Future works mainly includes the following parts: (1) Expanding the model to include other languages such as Persian; (2) using other word embedding methods to improve our model.

## Supplemental Information

10.7717/peerj-cs.877/supp-1 Supplemental Information 1 Sentiment dataset for Afghanistan (SD4A). We have presented a framework for data gathering and data sentiment labeling of twitter opinions. First, we considered tweets that were posted by users in the form of “Afghanistan” and “Afghan” hashtags to express their views about the current political situation in Afghanistan from 29/03/2018 to 21/06/2018. We then stored the retrieved tweets in the database and then labeled the dataset using the Vader dictionary.

## Additional Information and Declarations

### Data Availability

The following information was supplied regarding data availability: The computer codes are available at GitHub: https://github.com/alimarjan/ACR-SA-Main. The datasets are available at: - Glove word embedding is available at GloVe: Global Vectors for Word Representation: https://nlp.stanford.edu/projects/glove/ - IMDB Dataset of 50 K Movie Reviews: https://www.kaggle.com/lakshmi25npathi/imdb-dataset-of-50k-movie-reviews - The Sentiment dataset for Afghanistan (SD4A) dataset: Marjan Kamyab, “sentiment dataset for Afghanistan (SD4A).” Kaggle, 2021, DOI 10.34740/KAGGLE/DSV/2380237. - Sentiment140: http://help.sentiment140.com/for-students - Twitter US Airline Sentiment: https://www.kaggle.com/crowdflower/twitter-airline-sentiment Our model and other baseline implementation code for all four datasets are available at GitHub: https://github.com/alimarjan/ACR-SA-Main/tree/main/baselines.
