# Supplementary material for: ACR-SA: attention-based deep model through two-channel CNN and Bi-RNN for sentiment analysis
Source: PeerJ Comput Sci. 2022 Mar 17;8:e877. doi: 10.7717/peerj-cs.877 (PMC9044316; doi:10.7717/peerj-cs.877)
Supplement: Supplemental Information 1 — We have presented a framework for data gathering and data sentiment labeling of twitter opinions. First, we considered tweets that were posted by users in the form of “Afghanistan” and “Afghan” hashtags to express their views about the current political situation in Afghanistan from 29/03/2018 to 21/06/2018. We then stored the retrieved tweets in the database and then labeled the dataset using the Vader dictionary. [file peerj-cs-08-877-s001.zip › supplemental file/Sentiment dataset for Afghanistan (SD4A) data preparation processes..docx]

Sentiment dataset for Afghanistan (SD4A) data preparation processes.


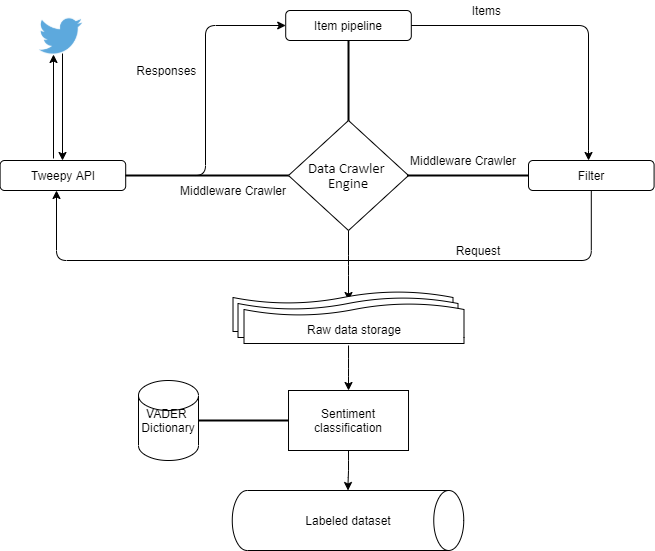


Figure 1 Module architecture for Twitter data gathering.

We have presented a framework for data gathering and data sentiment labeling of twitter opinions. First, we considered tweets that were posted by users in the form of “Afghanistan” and “Afghan” hashtags to express their views about the current political situation in Afghanistan from 29/03/2018 to 21/06/2018. We then stored the retrieved tweets in the database and then labeled the dataset using the Vader dictionary. The Module architecture is illustrated below in Figure 1. The frameworks steps as follows:

- Tweepy API: it is connecting the API with Twitter through Twitter developer authentication.
- Data Crawler Engine: It controls the data flow, launched by a Tweepy API, through all the components.
- Item Pipeline: It deals with the received content by cleansing, verifying, and storing it.
- Filter: it requests data based on the keywords and receives items in a sequential queue.
- Raw data Storage: store the raw datasets.
- Sentiment classification: it classifies the data using the Vader lexicon dictionary.
- Middleware crawler: It is a specific hook for dealing with the input and output of each item.
- Labeled dataset: finally, the labeled dataset is ready for training.

Table 2 Detailed statistics of the SD4A dataset.

| Dataset | Positive | Negative | Total |
| --- | --- | --- | --- |
| SD4A | 18309 | 18539 | 36848 |
